# Mortality trend analysis of ischemic heart disease in China between 2010 and 2019: a joinpoint analysis

**DOI:** 10.1186/s12889-023-15549-3

**Published:** 2023-04-04

**Authors:** Xiaoli Fu, Jing Wang, Shuai Jiang, Jian Wu, Zihan Mu, Yanyu Tang, Suxian Wang, Hang Fu, He Ma, Yaojun Zhao

**Affiliations:** 1 Department of Health Management of Public Health, Zhengzhou University, Henan, People’s Republic of China; 2 The First Affiliated Hospital of Zhengzhou University, Henan, People’s Republic of China; 3 Operation Management Department, Central China Fuwai Hospital, Central China Fuwai Hospital of Zhengzhou University, Henan, People’s Republic of China; 4 Institute for Hospital Management of Henan Province, Henan, People’s Republic of China; 5 Health Service and Management, Shangzhen College, Henan University of Traditional Chinese Medicine, Henan, People’s Republic of China

**Keywords:** Ischemic heart disease, Mortality rate, Mortality trend

## Abstract

**Background:**

This study presented the mortality trend of ischemic heart disease (IHD) in Chinese residents from 2010 to 2019 and provided a basis for further establishment of relevant interventions.

**Methods:**

Data, such as sex, age, urban and rural areas, and death status, were extracted from the China Death Surveillance Dataset from 2010 to 2019, with mortality and age-adjusted death rates (AADRs) as the main indicators. The joinpoint regression model was used to analyze mortality and AADRs trends in IHD. A semi-structured expert interview was conducted to propose targeted intervention measures and countermeasures.

**Results:**

We observed an overall upward trend in IHD mortality rates and AADRs in China from 2010 to 2019 (average annual percentage change [AAPC] = 5.14%, AAPC_AADRs_ = 1.60%, P < 0.001). Mortality rates and AADRs increased for both males (AAPC = 4.91%, AAPC_AADRs_ = 1.09%, P < 0.05) and females (AAPC = 5.57%, AAPC_AADRs_ = 1.84%, P < 0.001), with higher mortality rate for males than females but higher variation for females than males. Urban (AAPC = 4.80%, AAPC_AADRs_ = 1.76%, P < 0.05) and rural (AAPC = 5.31%, P < 0.001; AAPC_AADRs_ = 0.99%, P > 0.05) mortality rates increased, with the mortality rate higher in rural areas than in urban areas. In the age analysis, mortality rate was higher in middle-aged and older adults than in other age groups. The age-sex cross-analysis found the highest trend in mortality rates among females aged ≥ 75 years (annual percentage change [APC] = 2.43%, P < 0.05).

**Conclusions:**

The upward trend in IHD mortality in China from 2010 to 2019, especially among female residents aged ≥ 75 years, poses continuing challenges to public health policies and actions.

**Supplementary Information:**

The online version contains supplementary material available at 10.1186/s12889-023-15549-3.

## Background

Ischemic heart disease (IHD) is a type of heart attack caused by myocardial ischemia as a result of narrowing or blockage of blood vessels that supply the heart [1, 2]. IHD is the most fatal disease [3, 4] and the leading cause of health loss from cardiovascular disease (CVD) worldwide [5], especially in low- and middle-income countries [6]. Although the mortality rate of IHD has decreased in high-income countries, it remains the leading cause of death [1, 7].

A total of 197 million cases of IHD were reported in 2019 worldwide, with 9.14 million deaths from the disease. The total number of disability-adjusted life years (DALYs) due to IHD has steadily increased since 1990, reaching 182 million DALYs in 2019 [4]. IHD mortalities since 2017 have ranked second and are the leading cause of increased DALYs in the 50–74 and 75–year and older age groups [8]. IHD is the second leading cause of death in China, which results in a great economic burden to social and economic development. China’s age-adjusted death rate (AADR) from IHD has increased steadily since the 1990s, increasing by 20.6% in 2017 [9].

Current studies on IHD at national and international levels are more focused on the pathogenesis, medical techniques, and other clinical issues of IHD [10–12]. In Xuzhou, China, no significant changes in IHD were observed from 2011 to 2015; in 2011 and 2015 the mortality rates were 117.1/100,000 (APC = −1.0%, P > 0.05) [13]. Studies on the impact of air pollution on IHD in Wuhan Province and the burden of low-fiber diet on IHD in China from 1990 to 2017 have shown an increasing trend in IHD mortality in recent years. The mortality trends of IHD, including local and individual risk factors, in China have been widely studied [9, 13–15],but data for larger regions or countries are insufficient. According to Healthy China 2030, the control and prevention of CVD have become important tasks as the incidence and mortality rates of CVD continue to increase [16]. China’s medium- and long-term plans for the prevention and treatment of chronic diseases (2017–2025) call for a 15% reduction in the mortality rate of CVD by 2025 compared with 2015. Through education for all in the prevention and treatment of chronic diseases, China is continuously improving the health literacy of the population and conducting screening and intervention for chronic diseases, including CVD. The prevention and treatment of CVD, such as IHD, have become urgent public health issues. To determine the mortality trend of IHD in China, provide further scientific evidence for CVD prevention and treatment, and explore trends in mortality for continued improvement of public health interventions, we analyzed the trend of IHD mortality rates and AADRs for sex, urban and rural areas, and age using Death Surveillance Dataset data from 2010 to 2019 across China. China started the new medical reform in 2009, and 2010 was the first year after the reform. Therefore, we chose 2010 as the cut-off point to study the change in trend of IHD mortality rates after the new medical reform.

## Methods

### Data sources

**Fig. 1:**
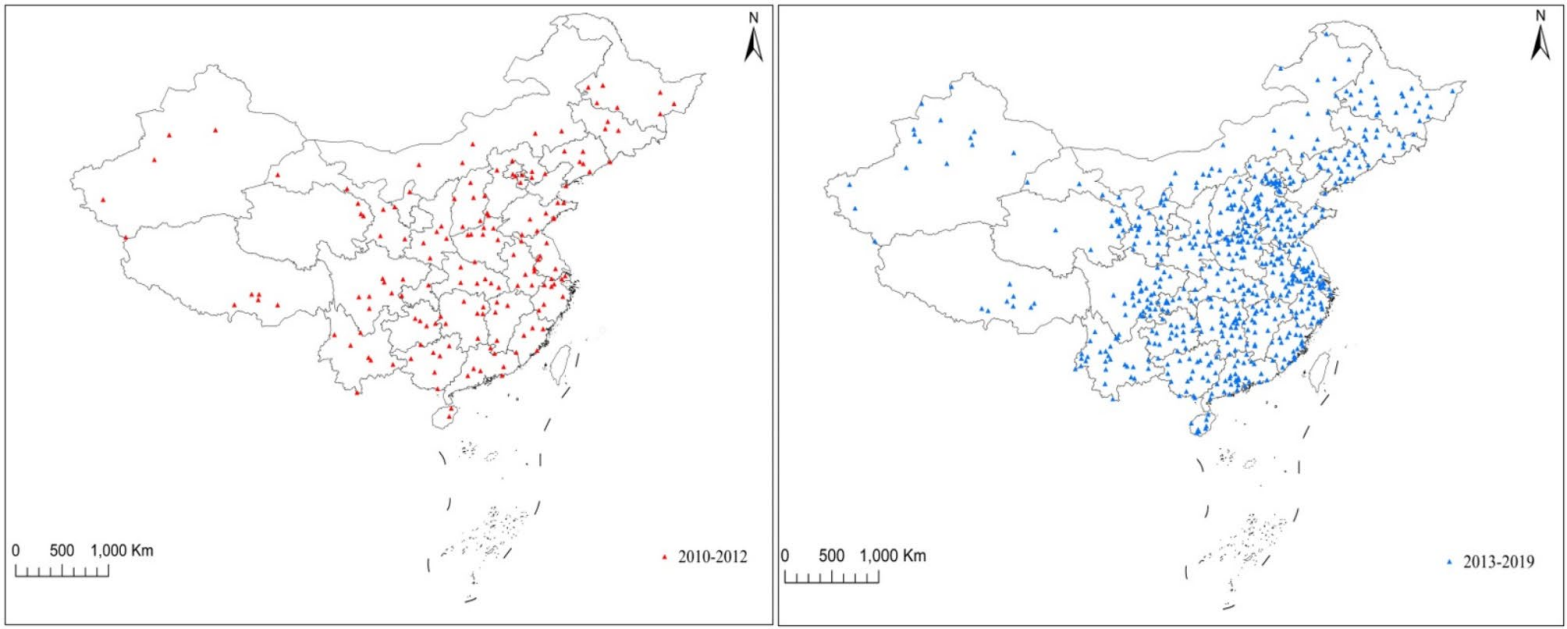
The map of surveillance points in different years

The study was based on the China Death Surveillance Dataset from 2010 to 2019. The National System of Disease Surveillance Points was established in 1978. In 1989, the number of surveillance points increased to 71, covering 29 provinces (autonomous regions and municipalities directly under the central government), officially forming the Disease Surveillance Points (DSP) system. In 1990, a new DSP system was established in 31 provinces (autonomous regions and municipalities directly under the central government), based on the principle of multistage, stratified cluster sampling, to cover 10 million people under surveillance. In 2003, adjustments were made based on systematic evaluation, including 161 monitoring points in 31 provinces (autonomous regions and municipalities directly under the central government), with a total monitoring population of more than 73 million, covering 6% of the population. In 2013, the former Ministry of Death Statistics System, DSP system, and other death reporting systems were integrated and expanded to establish a China Death Surveillance System. As a result of this integration, the number of monitoring points has expanded to 605, covering more than 300 million people, accounting for 24% of the country’s population. Details of the monitoring points are shown in Fig. 1.

### Data collection and quality

The China Death Surveillance System registers all deaths occurring in various jurisdictions, including Chinese residents, both registered and non-registered, and Hong Kong, Macao, Taiwan, and foreign citizens. All deaths in the China Death Surveillance System are reported online through the China Centers for Disease Control and Prevention’s Death Registration Reporting Information System. China’s Centers for Disease Control and Prevention reviews data reported by provinces and verifies and corrects any problems identified.

Omission is unavoidable; therefore, data from monitoring points that are seriously underreported and potentially affect the overall outcome are eliminated. From 2010 to 2012, the monitoring point mortality rate was below 3‰ as the exclusion criterion, and the minimum mortality rates at the monitoring points were 3.27‰, 3.07‰, and 3.05‰, respectively. From 2013 to 2019, the former Ministry of Health’s death statistics system and National Disease Surveillance System’s surveillance sites used the mortality rate below 4.5‰ as the exclusion criterion. The new surveillance sites added in 2013 used 5‰ as the exclusion criterion. The lowest mortality rates included in the analysis in the last 7 years ranged from 4.50‰ to 4.59‰.

### Statistical analysis

IHD (I20–I25) (angina pectoris, acute myocardial infection, subsequent myocardial infection, chronic IHD) was classified and coded according to the International Statistical Classification of Diseases and Related Health Problems (ICD-10). Data from the dataset for fields, such as sex, age, urban and rural areas, and death status, were selected. Data were collected and analyzed using the Statistical Package for the Social Sciences (SPSS) (version 20.0) and other software. Mortality and AADRs of IHD were used as the main analysis indicators. Joinpoint regression model (version.4.8.0.1) was used to calculate AADRs.

An AADR is a weighted average of mortality rates, where the mortality rates are calculated for different age groups and the weights are the proportions of persons in the corresponding age groups of a standard population. The AADR for an age group comprised of the ages *x* through *y* is calculated using the following formula.

$$\eqalign{&{AADR}_{x-y} =\cr& \sum _{i=x}^{y}\left[\left(\frac{{count}_{i}}{{pop}_{i}}\right)\times \text{100,000}\times \left(\frac{{stdpop}_{i}}{{\sum }_{i=x}^{y}{stdpop}_{i}}\right)\right]}$$

Where count is the number of cases in the *i*th age group, pop_*i*_ is the relevant population for the same age group, and stdpop_*i*_ is the standard population for the same age group.

The chi-squared test was used to compare the mortality rate among different groups. The trend in IHD was calculated from mortality rates and AADRs using the joinpoint regression model for the total surveillance population, male/female categories, urban and rural areas, and age groups. The default number of joinpoint was 0 or 1. The analysis started from 0 joinpoints and tests, and whether it was necessary to input one or more connection points in the model again to compare the models with the best fitting data was assessed. Subsequently, the most concise and suitable model to report the estimated annual percentage change (APC) detected in each period and the average annual percentage change (AAPC) at only 0 joinpoints or throughout the study period was selected. When APC or AAPC was statistically significantly different from 0, the term increase or decrease to describe the trend was used; otherwise, the term stable was used. All significance tests were two-sided. Statistical significance was defined as P < 0.05.

APC helps characterize trends of morbidity and prevalence over time. When the Log Transformation option on the Input File tab is *ln(y) = xb*, then the output calculates the estimated APC. Rates that change at a constant percentage annually show a linear change on a log scale. For this reason, to estimate the APC for a series of data, the following regression model is used:

$log\left({R}_{y}\right)={b}_{0}+{b}_{1}y$ where $log\left({R}_{y}\right)$ is the natural log of rate in year $y$.

The APC from year $y$ to year $y+1=\left[\frac{{R}_{y+1}-{R}_{y}}{{R}_{y}}\right]\times 100$

$$=\frac{\left\{{e}^{{b}_{0}+{b}_{1}\left(y+1\right)}-{e}^{{b}_{0}+{b}_{1}\left(y\right)}\right\}}{{e}^{{b}_{0}+{b}_{1}\left(y\right)}}\times 100$$

$$=\left({e}^{{b}_{1}}-1\right)\times 100$$

One advantage of this approach of characterizing trends is the ability to compare measures across scales, for both rare and common diseases.

AAPC is derived by first estimating the underlying joinpoint model that best fits the data. The AAPC over any fixed interval is calculated using a weighted average of the slope coefficients of the underlying joinpoint regression line with the weights equal to the length of each segment over the interval. The final step of the calculation transforms the weighted average of slope coefficients to an APC. If we denote *b* as the slope *i* coefficient for each segment in the desired range of years, and *w* as the length of each segment in the range of years, then:

$${APC}_{i}=\left\{Exp\left({b}_{i}\right)-1\right\}\times 100$$

$$AAPC=\left\{exp\left(\frac{\sum {w}_{i}{b}_{i}}{\sum {w}_{i}}\right)-1\right\}\times 100$$

### Expert interview

To assess scientific and targeted measures, a semi-structured expert interview on how to effectively perform health intervention for people at high risk of IHD was conducted. The interviews were conducted after the data analysis. The expert inclusion criteria were: (1) researchers in universities, medical institutions, and health departments with more than 3 years of health-related research and practice; (2) intermediate or above professional title; and (3) independent thinking and willing to participate in the consulting work. We contacted 15 interviewees for interviews. Of these, four respondents were from the government health sector (26.7%), five from hospitals (33.3%), and six from universities (40.0%). Due to the location of the interviewees, online and offline interviews were combined. Fifteen interviews were conducted, including six online and nine offline, with two interviewees (lead interviewees and documentarians). The interviews had an average duration of approximately 30 (range, 15–60) mins. All experts had relevant academic backgrounds and worked in the fields of public health and medicine. To determine the interview outline, we consulted relevant literature and sought the opinions of experts. (See Appendix 1 for details of the interview outline).

## Results

### Analysis of death from ischemic heart disease

IHD showed an overall upward trend in China from 2010 to 2019 (AAPC = 5.14%, AAPC_AADRs_ = 1.60%, P < 0.001). Mortality rates and AADRs were higher in males than in females, with statistically significant differences (P < 0.001). The number of males dying from IHD was consistently higher than that of females over a 10-year period, with approximately 15% more males dying each year on average than females. Mortality rates and AADRs increased in males (AAPC = 4.91%, AAPC_AADRs_ = 1.09%, P < 0.05) and females (AAPC = 5.57%, AAPC_AADRs_ = 1.84%, P < 0.001). The mortality rates and AADRs increased for both males and females, but the increase was lower for males than for females.

**Fig. 2:**
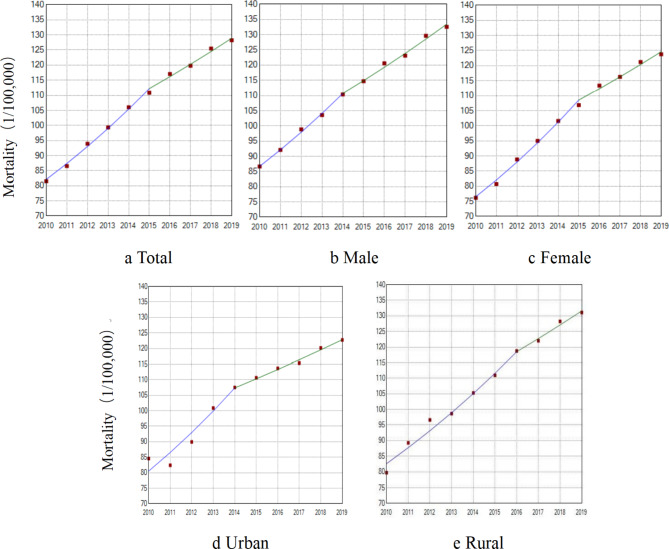
Trend of mortality of IHD from 2010 to 2019 by sex and urban/rural areas (/100,000) a) Total: 2010–2015, APC = 6.44%^*^; 2015–2019, APC = 3.53%^*^ AAPC = 5.14%^*^ b) Male: 2010–2014, APC = 6.29%^*^; 2014–2019, APC = 3.81%^*^ AAPC = 4.91%^*^ c) Female: 2010–2015, APC = 7.23%^*^; 2015–2019, APC = 3.52%^*^ AAPC = 5.57%^*^ d) Urban: 2010–2014, APC = 7.45%^*^; 2014–2019, APC = 2.73%^*^ AAPC = 4.80%^*^ e) Rural: 2010–2016, APC = 6.20%^*^; 2016–2019, APC = 3.55%^*^ AAPC = 5.31%^*^ Note: ^*^The estimated regression model had statistical significance

**Fig. 3:**
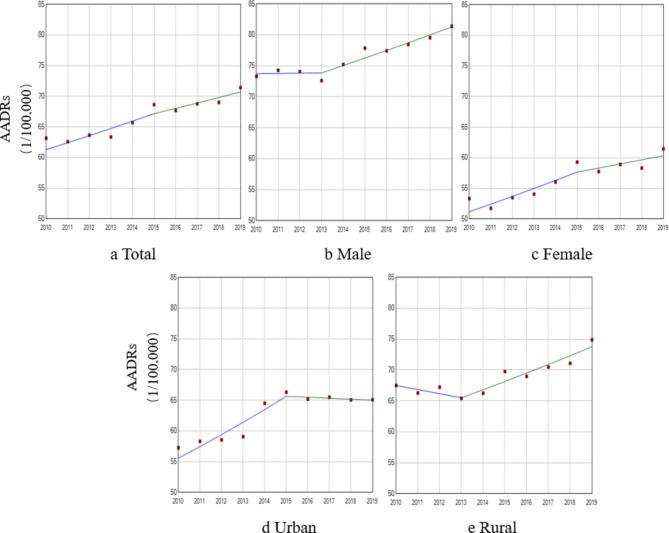
Trend of AADRs of IHD from 2010 to 2019 by sex and urban/rural areas (/100,000) a) Total: 2010–2015, APC = 1.84%^*^; 2015–2019, APC = 1.29%^*^ AAPC = 1.60%^*^ b) Male: 2010–2013, APC = 0.06%; 2013–2019, APC = 1.61%^*^ AAPC = 1.09%^*^ c) Female: 2010–2015, APC = 2.41%^*^; 2015–2019, APC = 1.14% AAPC = 1.84%^*^ d) Urban: 2010–2015, APC = 3.39%^*^; 2015–2019, APC = − 0.25% AAPC = 1.76%^*^ e) Rural: 2010–2013, APC = − 0.99%; 2013–2019, APC = 2.00%^*^ AAPC = 0.99% Note: ^*^The estimated regression model had statistical significance

The mortality rates of IHD among urban and rural residents has been increasing annually. Urban mortality rates and AADRs showed an upward trend (AAPC = 4.80%, AAPC_AADRs_ = 1.76%, P < 0.05); rural mortality rates showed an upward trend (AAPC = 5.31%, P < 0.001). The change in urban mortality rate was lower than that in rural mortality rate (Figs. 2 and 3). In addition, our analysis of urban and rural areas by sex was consistent with the overall trend.

### Mortality changes of ischemic heart disease in different age groups

**Fig. 4:**
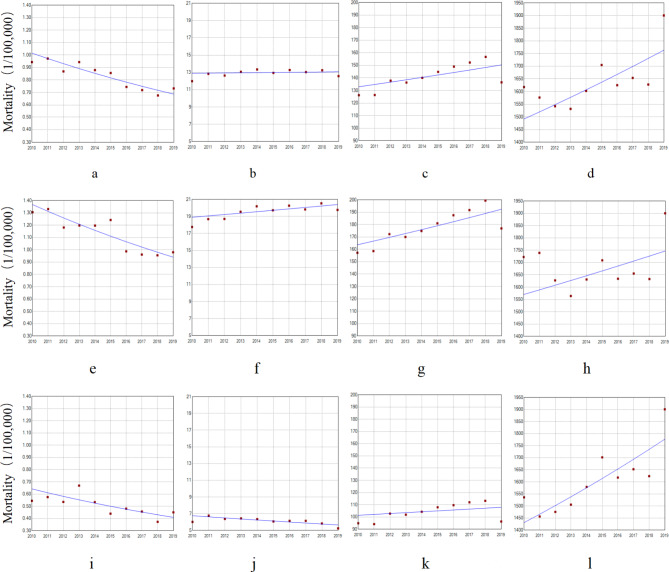
Trend of mortality of IHD from 2010 to 2019 by sex and age (1/100,000) 10–24: a) total: APC = − 4.25%^*^, e) male: APC = − 4.07%^*^, i) female: APC = − 4.88%^*^ 25–49: b) total: APC = 0.15%, f) male: APC = 0.85%^*^, j) female: APC = − 1.96%^*^ 50–74: c) total: APC = 1.37%, g) male: APC = 1.81%^*^, k) female: APC = 0.70% ≥ 75: d) total: APC = 1.88%^*^, h) male: APC = 1.19%, l) female: APC = 2.43%^*^ Note: ^*^The estimated regression model had statistical significance

Overall, the mortality rate for the 10–24-year age group showed a downward trend (APC = − 4.25%, P < 0.001), and an upward trend was observed for the 75-year and older age group (APC = 1.88%, P = 0.027). From a male perspective, there was a downward trend in the population aged 10–24 years (APC = − 4.07%, P = 0.001), but an upward trend in the mortality rate for the population aged 25–49 (APC = 0.85%, P = 0.035) and 50–74 years (APC = 1.81%, P = 0.022) was observed. From a female point of view, the mortality rate for the 10–24-year (APC = − 4.88%, P = 0.013) and 25–49-year (APC = − 1.96%, P = 0.009) age groups showed a downward trend, but an upward trend for the 75-year and older age group was observed (APC = 2.43%, P = 0.007). Since the number of deaths in the 0–9-year age group in multiple years was 0, trend analysis was not conducted (Fig. 4).

### Age–sex cross-analysis

**Fig. 5:**
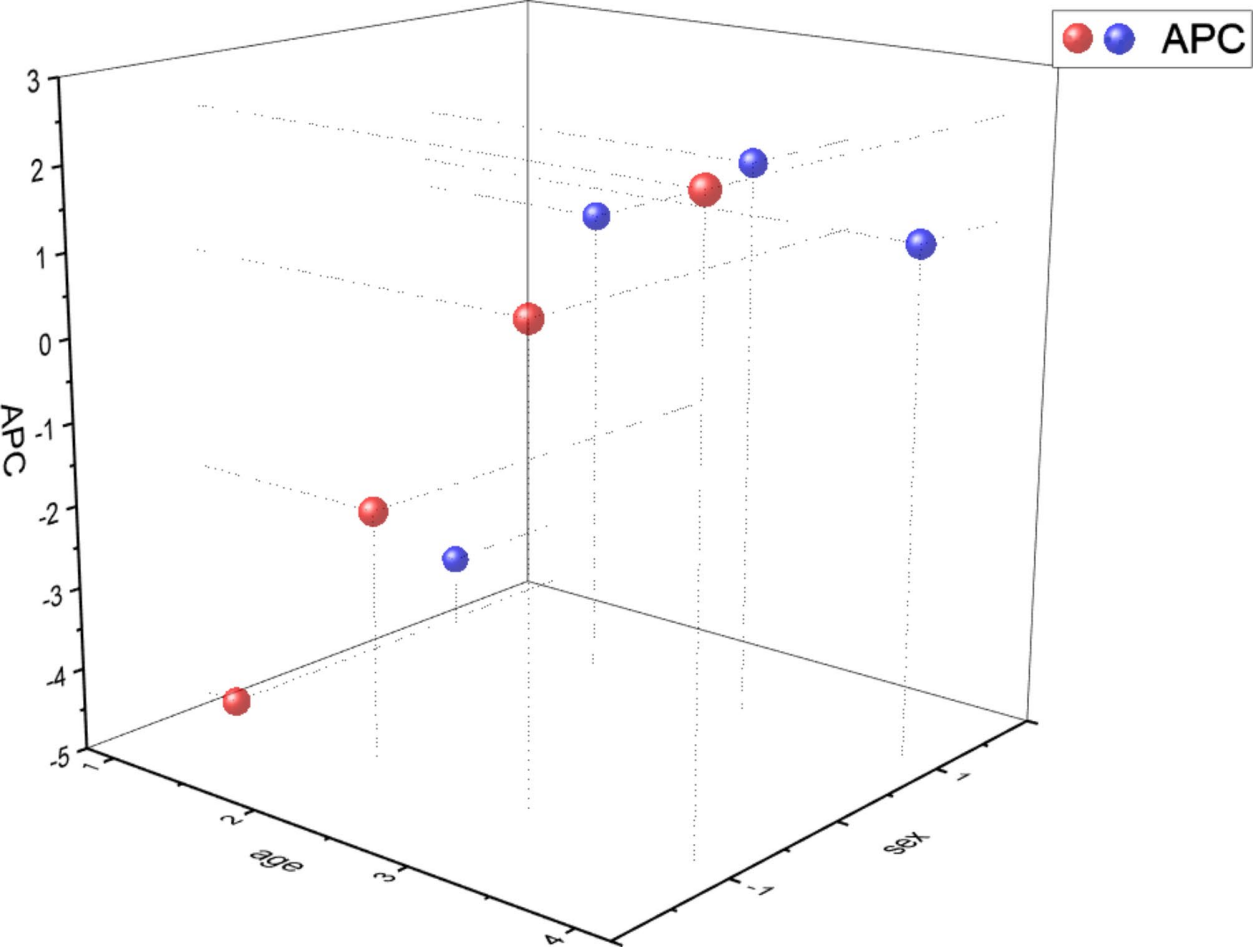
Cross-analysis of age and sex Note: sex: 1 = male, − 1 = female age: 1 = 10–24, 2 = 25–49, 3 = 50–74, 4 = ≥ 75

Cross-analysis by age group and sex found an increase in the variability of IHD mortality rate with age. The increase was highest among females aged ≥ 75 years (APC = 2.43%), followed by males aged 50–74 years (APC = 1.81%) (Fig. 5).

### Summary of Expert interview

We analyzed the data of semi-structured interviews and summarized the main contents of countermeasures and suggestions by keywords. (Appendix 2)

In C1, 86.67% (13/15) of the respondents believed that the increasing trend of IHD mortality rate was related to risk factors, such as the cardiovascular effects of adverse lifestyle conditions, including smoking.

In C2, apart from lifestyle differences, 46.67% (7/15) of the respondents mentioned differences in physiological functions between males and females as reasons for the sex differences in IHD. All participants (15/15) stated that the difference in mortality rates between rural and urban areas is mainly due to discrepancies in medical care between urban and rural areas. The participants agreed on the need to expand the scope of primary health care and to raise awareness of IHD through health education and disease screening for early diagnosis and treatment.

In C3, 73.34% (11/15) of the respondents considered that the current high mortality trend in females was due to society’s underestimation of female’s risk and that clinical studies of IHD in female should be strengthened.

In C4, 93.34% (14/15) of the respondents stated that physical skills decline as people get older. All participants (15/15) suggested that the elderly should improve their physical fitness through physical exercise and should be encouraged to undergo regular health check-ups.

In C5, 60.00% (9/15) of the respondents believed that the mortality trend in females was related to changes in body function and hormone levels after menopause. Of note, 80.00% (12/15) of the respondents recommended more focus on the elderly females over 75 years old and targeted screening for cardiovascular diseases.

## Discussion

We analyzed the mortality trend of IHD in China from 2010 to 2019. The aim is to understand the disease burden of IHD in China in a comprehensive manner to provide targeted prevention and control of the disease, improve the current situation of health management, improve the quality of life of Chinese residents, formulate corresponding public health policies, and provide data support and evidence-based information.

The increasing trend of IHD mortality in China was significantly related to various risk factors. Smoking, diabetes, high blood pressure, high serum cholesterol level, and physical inactivity are major risk factors for IHD. A Japanese study showed that smoking had a risk ratio (RR) of 2.21 (95% confidence interval [CI]= (1.96–2.50)) [17], and hospitalizations and mortality rates from IHD were significantly higher in smokers compared to nonsmokers [18, 19]. Second-hand smoke also has a significant effect on IHD [20], and smoking bans in public places are effective in reducing hospitalization rates for IHD [21, 22]. Diabetes independently induces myocardial dysfunction and structural abnormalities [23], increasing ischemic heart load, which consequently raises the incidence of IHD [24, 25]. Controlling blood pressure and cholesterol levels can reduce the risk of IHD by at least 16%, according to the World Health Organization. Changes in the Chinese diet, which has become dominated by high fat and calorie intake, have led to higher serum cholesterol levels in the population, leading to higher incidence and mortality rates of IHD. Meat intake has also been associated with higher rates of IHD [26]. In addition, a previous study showed a significantly increased risk of IHD among physically inactive participants [27].

The number of deaths and mortality of males with IHD were higher than those of females, consistent with the results of multiple studies[3, 13, 28, 29], suggesting that male residents are more affected by IHD than female residents. In addition, male deaths from IHD are concentrated in the middle-aged and older age groups. The higher male mortality rate may be due to a combination of factors, such as higher male smoking rate (47.6%) than female smoking rate (1.9%) [30] and higher weighted male hypertension rate (24.5%) than weighted female hypertension rate (21.9%)[31]. As shown in a previous Korean study, females with hypertension have a lower risk of IHD than males (hazard ratio [HR] = 0.93) [32]. In conjunction with the expert group interviews, health education should be directed at male residents to raise awareness of self-care and the prevention and treatment of CVD, such as IHD. As a preventable and controllable cause of death, the government should strengthen tobacco control campaigns to reduce the health harm caused by smoking. For patients with hypertension, regular health screenings are recommended to achieve early detection, diagnosis, and treatment to minimize the risk of IHD.

Mortality from IHD increased more in females than in males. Several studies have shown that females are more likely to die from IHD than males [33, 34]. Differences in pathology and clinical presentation, delays in diagnosis and treatment, and underrepresentation of females in clinical trials contribute to these results [35]. We may have underestimated the risk of IHD in females. Studies have shown that females with diabetes have a four- to six-fold increased risk of IHD [34], and that the burden of physical activity-induced IHD is higher for females than for males [36]. In addition to these several risk factors, females have distinct risk factors, including pregnancy-related complications and ovarian disease [37]. The incidence rate of gestational diabetes has increased in recent years due to the obesity epidemic and the increase in the reproductive age. Studies have shown that cardiovascular risk increases for females with gestational diabetes, preeclampsia, and eclampsia [38]. Increasing attention to CVD, such as IHD, in females, moving away from male-centered diagnosis and management models, and strengthening sex-specific stratification of cardiovascular risk factors are considered significant strategies for females at high risk of CVD. Health education for women at high risk should also be provided for timely diagnosis and treatment.

The IHD mortality rate was higher among rural residents than urban residents, which is consistent with the results of a previous study [39]. The rural-urban disparities are attributable to the inadequate level of medical care in rural areas, lack of medical facilities, and high prevalence of related risk factors [39, 40]. Rural health resources are less than urban resources, which hinders proper primary health care in rural areas [41] and constitutes an obstacle to improving the health status of the population. In addition, rates of overweight and obesity have increased rapidly in rural areas [42], and rural populations have higher rates of smoking [30] and hypertension than urban populations [31]. Therefore, the government should increase rural health investment, reduce urban and rural inequities, encourage graduates to work at the grassroots level, strengthen the re-education of medical personnel, and continuously improve the standards of medical personnel at the grassroots level. Health policymakers can alleviate rural-urban disparities in health care and disease outcomes by understanding the specific needs of rural areas and developing and implementing prevention strategies for CVD, such as IHD, based on rural settings. We will continue to improve primary health care for CVD, raise the awareness of rural residents regarding relevant risk factors, and reduce the mortality rate of IHD.

Age analysis showed that older adults were more likely to die from IHD, with the majority deaths occurring in people aged ≥ 75 years, consistent with findings from several studies [28, 43]. Aging is a risk factor for CVD, and with age, coronary and microvascular functions and adaptability decrease and can alter myocardial perfusion damage [10]. The mortality rate of IHD in the elderly has been increasing annually, which may be related to the deepening aging of the population. Aging is the leading cause of several chronic diseases. As of 2019, China had approximately 160 million citizens aged ≥ 65 years, 15% of whom were aged ≥ 80 years [44]. Therefore, it is urgent to realize a healthy aging society in the face of the increasing level of aging and the serious medical and socioeconomic challenges. Experts recommend tobacco control campaigns aimed at the elderly; promoting a reasonable and balanced diet; reducing the intake of foods high in oil, fat, and sodium; eating vegetables, grains, eggs, fish, and other high-protein foods; and actively participating in physical exercise. Particularly, people with dyslipidemia need to boost their health by improving their diet and physical activity. Regular medical check-ups are encouraged for early detection, active control, and management of family history of high-risk conditions, such as high blood pressure, high blood fat level, diabetes, obesity, and coronary heart disease.

An interesting finding was that in cross-analysis of age and sex, the highest increase in mortality was observed among females aged ≥ 75 years. Studies have shown that females are more likely to develop IHD in later life than males [34], with atypical symptoms [35]. Females typically develop IHD 10 years later than males. Females lose the protective effects of estrogen after menopause, and the risk of CVD increases dramatically [35].Expert group interviews recommended focused screening of females aged ≥ 75 years to control exposure to risk factors, with particular attention to the presence of atypical symptoms of IHD. People at high risk of CVD are advised to have regular blood fat, blood pressure, and heart function tests to monitor their health status and to exercise properly.

### Limitations

At present, there are no universal standard for determining the main causes of death from IHD in the world. The use of different criteria for diagnosis causes differences in prevalence rates and mortality from IHD. Even within the same country, such criteria depend both on the scientific schools in certain regions and on the resource capacity of doctors and hospitals. These factors can significantly alter the mortality rates. IHD is often associated with a variety of complications, making it difficult to determine the underlying cause of death. The data analyzed in the present study may not accurately represent the mortality rate of IHD. In addition, the effect of regions, provinces, and different levels of economic development on mortality rates was not considered in the analysis of trends in IHD mortality.

## Conclusions

By analyzing the mortality rate of IHD in China from 2010 to 2019, this study concludes that the mortality rate of IHD is increasing and is higher for males than for females, but with higher variation for females than for males, with the highest increase in death rate among females aged ≥ 75 years. These reflect changes in lifestyle and habits, such as lack of physical activity, high cholesterol level, and smoking. Therefore, controlling the risk factors for IHD to reduce morbidity and mortality is an urgent matter in China.

## Electronic supplementary material

Below is the link to the electronic supplementary material.

Supplementary Material 1

## Data Availability

All data generated or analyzed during this study are included in this published article.

## Abbreviations

IHD: Ischemic Heart Disease
AADRs: Age-Adjusted Death Rates
CVD: Cardiovascular Diseases
DALY: Disability-Adjusted Life Year
ICD-10: International Classification of Disease 10
DSP: Disease Surveillance Point
APC: Annual Percentage Change
AAPC: Average Annual Percentage Change
