# Supplementary material for: Mortality trend analysis of ischemic heart disease in China between 2010 and 2019: a joinpoint analysis
Source: BMC Public Health. 2023 Apr 4;23:644. doi: 10.1186/s12889-023-15549-3 (PMC10071740; doi:10.1186/s12889-023-15549-3)
Supplement: Supplementary file 1 — Supplementary Material 1 [file 12889_2023_15549_MOESM1_ESM.docx]

**Appendix**

**Interview outline**

**Interview purpose**

Based on the findings of this study, we conducted a semi-structured open expert interview on how to effectively implement health interventions for people at high risk of ischemic heart disease（IHD）. The purpose of this study is to provide scientific advice to the government and relevant health authorities on effective interventions to reduce the prevalence and mortality of IHD and improve the health status of the population.

**Interview time**

July 1st to July 15th, 2022.

**Interview method**

Interviews were conducted online and offline, mainly in person, with an average of 30 minutes. Interviews were conducted in a semi-structured question-and-answer format, with two interviewees (primary interviewees and documentaries), both with backgrounds in public health and preventive medicine.

Before the interview began, we explained the purpose of the interview to the interviewed experts and recorded the interview in full with their prior consent.

During compiling the interview materials, we numbered the interviewed experts, and based on verbatim transcriptions, we standardized the oral narratives of the interviewed experts to remove oral language and meaningless repetitions from the interview narratives.

**Interview content**

Appendix 1. Interview Content

| Category | Questions |
| --- | --- |
| 1. Basic Information | A1. What is your age? |
|  | A2. What is your nationality? |
|  | A3. What is your highest level of education? |
|  | A4. What is your professional title? |
| 2. Field of Work | B1. What is your main research direction? |
|  | B2. What role do health promotion and interventions play in your daily routine? Please describe an example. |
| 3.Countermeasures and Suggestions | C1. What do you think of the rising death rate from IHD in China from 2010 to 2019? |
|  | C2. What is your understanding and intervention suggestion on the mortality rate of IHD in China from 2010 to 2019, which is higher for men than for women and higher for rural residents than urban residents? |
|  | C3. What is your understanding of the trend of higher mortality rate of IHD among women than among men in China from 2010 to 2019 and your intervention suggestions? |
|  | C4. What do you know about the higher death rate from IHD in middle-aged and older adults and recommendations for interventions? |
|  | C5. What is your understanding of, and intervention recommendations for, the highest mortality variability among female residents aged 75 and older? |

Appendix 2. Summary of the Main Contents of Countermeasures and Suggestions in the Interview

| Questions | Key words |
| --- | --- |
| C1. What do you think of the rising death rate from IHD in China from 2010 to 2019? | major causes of death;  associated with risk factors; |
| C2. What is your understanding and intervention suggestion on the mortality rate of IHD in China from 2010 to 2019, which is higher for men than for women and higher for rural residents than urban residents? | male smoking and other lifestyle habits;  physiological differences between men and women;  differences in medical care between urban and rural areas;  disease screening;  health education;  improve primary health care; |
| C3. What is your understanding of the trend of higher mortality rate of IHD among women than among men in China from 2010 to 2019 and your intervention suggestions? | risk factors unique to women;  underestimating women's risk;  strengthening IHD studies in women;  screening for diseases; |
| C4. What do you know about the higher death rate from IHD in middle-aged and older adults and recommendations for interventions? | the decline in physical function associated with aging;  aging is deepening;  controlling high risk factors;  early screening of diseases;  medical examination;  strengthen body; |
| C5. What is your understanding of, and intervention recommendations for, the highest mortality variability among female residents aged 75 and older? | changes in body function after menopause;  encourage research;  pay attention to the health of the population;  carry out key screening; |
